# Circ_0004354 might compete with circ_0040039 to induce NPCs death and inflammatory response by targeting miR-345-3p-FAF1/TP73 axis in intervertebral disc degeneration

**DOI:** 10.1155/2022/2776440

**Published:** 2022-01-07

**Authors:** Yongjin Li, Xiaojing Wu, Jianhua Li, Lilong Du, Xuke Wang, Jiasong Cao, Hao Li, Zhenxin Huo, Guowang Li, Dayu Pan, Haiwei Xu, Baoshan Xu

**Affiliations:** ^1^Department of Minimally Invasive Spine Surgery, Tianjin Hospital, 406. No, Jiefangnan Road, Hexi district, Tianjin, China 300211; ^2^Graduate School, Tianjin Medical University, 22 Qixiangtai Road, Tianjin 300070, China; ^3^Department of Surgery Critical Care Medicine, Beijing Shijitan Hospital, Capital Medical University, No. 10 Tieyi Road, Haidian District, Beijing 100038, China; ^4^Department of Orthopaedics, Tianjin Haihe Hospital, Tianjin 300350, China; ^5^Department of Minimally Invasive Spine Surgery, Luoyang Orthopedic-Traumatological Hospital, QiMing Road, LuoYang city, China 471002; ^6^Tianjin key lab of human development and reproductive regulation, Tianjin Central Hospital of Obstetrics and Gynecology, Nankai University, Tianjin 300199, China

## Abstract

The abnormal function of nucleus pulposus cells (NPCs) plays a crucial role in the pathogenesis of intervertebral disc degeneration (IVDD). Recent studies have demonstrated that circular RNAs (circRNAs) are involved in the pathological process of IVDD by regulating NPCs' function. Nevertheless, the investigation on circRNA-circRNA interaction has not yet been reported. Here, we identified the top upregulated circ_0040039 and circ_0004354 in IVDD, derived from the syntrophin beta 2 gene but had different degrees of biological functions. Accumulating studies have reported PANoptosis is composed of apoptosis, pyroptosis, and necroptosis. Based on this, we think there should be a new pro-inflammatory cell death PAoptosis in the form of apoptosis and pyroptosis. Circ_0004354 might compete with circ_0040039 to induce the development of IVDD by modulating miR-345-3p-FAF1/TP73 axis-mediated PAoptosis, inflammatory response, growth inhibition, and ECM degradation of NPCs. Thus, these findings offer a novel insight into the circRNAs-mediated posttranscriptional regulatory network in IVDD, contributing to further clarification of the pathological mechanism of IVDD to develop a promising therapeutic target for IVDD diseases.

## 1. Introduction

In 2019, the analysis of the global burden of diseases identified low back pain (LBP) as the predominant cause leading to dyskinesia in patients [1]. Intervertebral disc degeneration (IVDD) is one of the most critical contributors to trigger LBP [1–3], which is known to cause serious social-economic problems [1, 4]. The intervertebral disc (IVD) is a complex structure containing the central nucleus pulposus (NP) [5]. IVDD often begins with the degeneration of NP cells (NPCs). NPCs play a crucial role in supporting IVD structure and biological functions, as well as in maintaining IVD homeostasis by synthesizing extracellular matrix (ECM) components, especially aggrecan (ACAN) and collagen II alpha 1 (COL2A1, COL2) [6, 7]. Inflammatory cell death, encompassing NPCs apoptosis and pyroptosis, also influences the biological functions of IVD [8, 9]. The elevated secretion of proinflammatory cytokines, especially tumor necrosis factor (TNF)-*α* and interleukin (IL)-1*β*, is another vital trait of the degeneration of the NPCs [2, 3, 10]. During IVDD, the developing inflammation presents a waterfall-like cascade reaction, promoting hyperalgesia and nerve ingrowth, enhancing NPCs death and ECM breakdown [2, 3]. Thus, we hypothesized that the pro-inflammatory factors in the IVD microenvironment caused by various factors would continue to increase, and eventually form an inflammatory cascade, which is the key mechanism leading to LBP and aggravation of IDD [2, 3, 10]. Therefore, it was critical to conduct an in-depth investigation of the mechanism of inflammatory cell death in IVDD to find a novel method to eliminate inflammation and NPCs death.

Apoptosis is divided into the extrinsic and intrinsic apoptosis pathways, which is executed by the cleavage caspase3 (c-CASP3) [11–13]. Gasdermin E (GSDME) is the key executive protein of pyroptosis, which can be driven by CASP3 [13–15]. The occurrence of cells pyroptosis is accompanied by the secretion of IL-1*β* [13–15]. Since cells can undergo extensive crosstalk under pathological conditions, cell death usually does not occur independently but in a mixed form [11, 12]. Karki et al. [11] found that there was a mixed cell death PANoptosis composed of apoptosis, pyroptosis, and necroptosis. To facilitate the investigation of the relationship between cells death and inflammation, we think there should be a new pro-inflammatory cell death PAoptosis in the form of apoptosis and pyroptosis. However, the relationship between apoptosis and pyroptosis and the mixed death PAoptosis has not yet been reported in NPCs.

Circular RNAs (circRNAs), without a 5'-3' polarity and polyadenylation tail, are known to be highly resistant to RNase R [16]. Exonic circRNA is the most common type of circRNAs, which comes from the cyclization of at least one exon from a single gene [16, 17]. Exonic circRNAs is usually located in the cytoplasm, which mediate NPCs' functional changes by acting as competitive endogenous RNA (ceRNA) [7, 17–21]. microRNAs (miRNAs) can recognize and bind to the target gene's 3'-UTR by the seed sequence to degrade mRNA or inhibit translation by binding to argonaute 2 (AGO2) protein to form RNA-induced silencing complex (RISC) [22]. The dysregulation of the expressions of circRNAs and miRNAs act as a hallmark characteristic of IVDD. Increasing evidence has revealed that circRNAs and miRNAs play a crucial role in mediating the occurrence and progression of IDD through participating in the modulation of NPCs apoptosis, proliferation, inflammatory response, and ECM metabolism [7, 17–21]. However, these studies have focused on the regulation of miRNA by one circRNA; the impact of multiple circRNAs originating from the same parental gene has been neglected by the researchers. Furthermore, the role of circRNA-mediated NPCs pyroptosis is unclear. Thus, the goal of this study was to study the role and biological significance of multiple circRNAs in the IVDD progress.

Here, we obtained microarray datasets related to human lumbar NP tissues encompassing GSE67566 (circRNA), GSE63492/GSE116726 (miRNA), and GSE56081 (mRNA), from Gene Expression Omnibus database (http://www.ncbi.nlm.nih.gov/geo) [23] to perform bioinformatics analysis. We identified the top upregulated circ_0040039 and circ_0004354 in IVDD, originating from the syntrophin beta 2 (SNTB2) gene. Then, we performed an extensive analysis of their specific roles and underlying mechanisms *in vitro*.

## 2. Materials and methods

### 2.1. Ethics statement and NP tissues selection

This study was supervised and approved by the Tianjin Hospital Ethics Committee. All donors signed the informed consent form before surgery. We obtained 30 human degenerative NP tissues from patients with IVDD who were diagnosed with lumbar disc herniation, lumbar spinal stenosis, and lumbar spondylolisthesis and then conducted surgery on account of the severe LBP, neuralgia, or acute complications. The normal tissues were obtained from 16 patients with scoliosis, fresh thoracolumbar fracture, and spinal cord injury who were undergoing surgery due to spinal deformity, instability, or neurological deficits. Patients with rheumatoid arthritis, immune diseases, seropositive and negative spondyloarthropathy, thyroid diseases, tumors, and tuberculosis were excluded from the study. Supplementary Table 1 presents detailed information for each patient. The severity of IVDD was evaluated according to Pfirrmann's classification method [24]. Patients with Pfirrmann grade I/II were assigned to the normal group, whereas those with Pfirrmann grade III/IV constituted mild degeneration and grade V constituted the severe degeneration group. Supplementary Figure 1 shows the partial data on the patients' magnetic resonance imaging (MRI) information.

### 2.2. Analysis of circRNA, miRNA, and mRNA microarray datasets

CircRNA (GSE67566), miRNA (GSE63492/GSE116726), and mRNA (GSE56081) microarray datasets were downloaded from the GEO database [23] and analyzed by the limma package in R [25]. The screening criteria for differentially expressed circRNAs (DECs) was: -log_10_FDR>2 and |log_2_ fold-change (FC)| > log_2_5. The volcano plot of differentially expressed miRNAs (DEMs) was made according to the analysis of GSE116726 with the standard of FDR<0.05 and |log_2_ (FC)| >1. The upregulated mRNAs in GSE56081 were selected with the standard of *P*-value <0.01 and log_2_ (FC) >1.  Table 1 shows the detailed information for each dataset.

### 2.3. Bioinformatics analysis

The potential target miRNAs of key circRNAs were predicted using miRanda (http://www.microrna.org/microrna/home.do) [26], RNAhybrid (https://bibiserv.cebitec.uni-bielefeld.de/rnahybrid/submission.html) [27], Targetscan human 7.2 (http://www.targetscan.org/vert_72/) [28] databases, and GSE63492/GSE116726 datasets to select IVDD-related DEMs. Conversely, the upstream circRNAs of key miRNA were also predicted using the above databases and the GSE67566 dataset. The potential target genes of key miRNA were predicted by four programs, including Targetscan [28], starBase (http://starbase.sysu.edu.cn/index.php) [29], miRmap (http://mirmap.ezlab.org) [30], and mirDIP (http://ophid.utoronto.ca/mirDIP/) [31]. Then, the overlapping mRNAs were merged with the upregulated mRNAs of the GSE56081 dataset to further select IVDD-related DEGs. The screened DEMs and DEGs were used to construct circRNAs-miRNAs-mRNAs network using Cytoscape software version 3.7.1 [32]. Gene Ontology (Go) analysis was conducted through the Database for Annotation, Visualization, and Integrated Discovery (DAVID) tools (https://david-d.ncifcrf.gov/) [33] and visualized by Sangerbox tool (http://sangerbox.com/tool), a free online platform for data analysis. Furthermore, the clueGO plugin in Cytoscape software was applied to display TP73-mediated biological processes. *P*-value <0.05 was considered to be statistically significant. Additionally, a circRNA online database (https://circinteractome.nia.nih.gov/bin/circsearch Test) [34] was used to analyze the potential AGO2 protein binding sites of key circRNAs.

### 2.4. Acquirement, culture, and treatment of human NPCs

The precise method has been described in our previously study [20, 35]. We obtained the primary human NPCs from ScienCell Research Laboratories (Sciencell, Cat. #4800, USA). NPCs were cultured in NPC Medium (Sciencell, Cat. #4801, USA) containing 5 mL NPCs growth supplement, 5 mL penicillin/streptomycin solution, and 10 mL fetal bovine serum. They were incubated at 37°C in a humidified environment with 5% CO_2_. The medium was changed every 2 days, and the NPCs were passaged once a week. Then the well-grown NPCs were used in the following experiments. As described by previous studies, 10 ng/mL TNF-*α* and 10 ng/mL IL-1*β* (Peprotech) were used to treat NPCs^35^ or chondrocytes [36] for 24 h before experiments, so we used the same TNF-*α*/IL-1*β* concentration and treatment time to treat the NPCs after transfection of plasmids or miR-345-3p to simulate the micro-environment of IVDD *in vitro.*

### 2.5. Vectors construction and NPCs transfection

The empty vector: pcDNA3.1 + Circ Mini (5607 bp), as well as over-expression vector: pcDNA3.1 + Circ Mini-circ_0040039 (6333 bp) and pcDNA3.1 + Circ Mini- circ_0004354 (5765 bp) were designed and synthesized by Hy cell biotechnology (Wuhan, China). MiR-345-3p mimic and miR-345-3p inhibitor were ordered from Guangzhou Geneseed Biotech Co. (Guangzhou, China). The pcDNA3.1 + FAF1 (7337 bp) and pcDNA3.1 + TP73 were obtained from JIAMAY BIOLAB (Beijing, China). The effects of overexpression were detected by qRT-PCR and western blot experiments. Lipofectamine 8000 (Beyotime, China) was used to transfect plasmids or miR-345-3p or NCs into NPCs following the manufacturer's guidance. After 48 h transfection, NPCs were used to perform the subsequent functional identification experiments.

### 2.6. Quantitative real-time RT-PCR

The TRlzol Reagent (Life Technologies, Thermo Fisher Scientific, USA) was used to extract total RNAs from NPCs or NP tissues. To detect RNA concentration and purity, 1 *μ*L RNA sample was added to micro spectrophotometer (Nano-300, Allsheng, Hangzhou, China) and recorded the A_260_/_280_ ratio and RNA concentration. Then 1% agarose gel electrophoresis was performed using electrophoresis (Junyi, Beijing, China) to further identify RNA purity and integrity. Subsequently, we used 1 *μ*g total RNAs and 1 *μ*L Geneseed® Enzyme Mix (Geneseed, Guangzhou, China) to reverse into 20 *μ*L complementary DNA (cDNA) using Geneseed® II First Strand cDNA Synthesis Kit (Geneseed, Guangzhou, China). Subsequently, 10 *μ*L Geneseed® qPCR SYBR® Green Master Mix (Geneseed, Guangzhou, China), 0.5 *μ*L Forward (F) primer, and 0.5 *μ*L Reverse (R) primer were prepared to perform qRT-PCR on ABI 7500 system (Applied Biosystems, USA). Supplementary Table 2 shows all primers. CircRNAs and mRNAs expression was normalized to GAPDH, whereas miR-345-3p expression was normalized to U6. The relative expression levels of circRNAs, miRNA, and mRNAs were detected and analyzed using the 2^-△△^Ct method.

### 2.7. RNase R treatment and Actinomycin D (ACT-D) assay

The total RNAs (10 *μ*g) were extracted from NPCs and then incubated at 37°C for 30 min in the presence or absence of RNase R (Epicentre Technologies, Madison, WI, USA). Next, 2 mg/L ACT-D (Sigma-Aldrich, St. Louis, MO, USA) was used to treat NPCs at different time points to repress RNA synthesis. The relative expression of circ_0040039, circ_0004354, and SNTB2 mRNA were assessed through qRT-PCR.

### 2.8. Western blot analysis

RIPA lysis buffer containing phenylmethanesulfonylfluoride (Beyotime, Shanghai, China) was employed to extract proteins from NPCs. We then employed the Micro Bicinchoninic Acid Protein Assay kit (Beyotime, Shanghai, China) to quantify protein concentration. Next, the proteins were isolated through SDS-PAGE and then were transferred to polyvinylidene difluoride (PVDF) membranes (Millipore, Germany) at 350 mA for 90 min. Subsequently, the PVDF membranes were closed by 5% non-fat milk for 60 min and incubated overnight with primary antibody at 4°C and washed 10 min ×3 times using phosphate-buffered saline with Tween-20. Then the membranes incubation with a secondary antibody for 60 min at room temperature and followed by washing again. Finally, the PVDF membranes were put into a chemiluminescent substrate and developed for 2 minutes. Then the chemiluminescence system (Bio-Rad, CA, USA) was employed to detect the signals. Supplementary Table 3 shows the list of antibodies used in this study.

### 2.9. Cell Counting Kit-8 (CCK-8) and Flow cytometry (FCM)

The well-growth NPCs were inoculated into six-hole cell culture plates at a density of 5 × 10^5^ cells per well. Then, 200 *μ*L diluted RNAs-lipofectamine 8000 (Beyotime, China) complex were added to the cell wells that had been replaced with an 800 *μ*L of serum-free medium. The NPCs were then cultured for 0, 1, 2, and 3 days at 37°C incubators. For the CCK8 assay, 10 *μ*L CCK-8 solution was placed in each well and for incubation for another 1.5 h. The NPCs growth rate was evaluated by the CCK-8 detection kit (Yeasen, Shanghai, China) following the manufacturer's suggestions. The absorbance was determined at 450 nm. NPCs growth rates were calculated based on the formula: Day_n_ OD value/Day_0_ average OD value.

NPCs apoptosis rates were evaluated by Annexin V-FITC or Annexin V-APC apoptosis detection kit (keyGEN, Jiangsu, China). Annexin V-FITC was matched with propidium iodide (PI), and Annexin V-APC was matched with 7-AAD to distinguish NPCs in different stages of apoptosis. The NPCs were stained with 1.25 *μ*L Annexin V-FITC (or Annexin V-APC) and 10 *μ*L PI (or 7-AAD), and then the data were analyzed using Flowjo VX10 software. On the scatter plot of the bivariate FCM, (Q2) represented the late apoptotic and necrotic NPCs, whereas (Q3) represented the early apoptotic NPCs, so Q2 + Q3 reflected the apoptosis rate of NPCs.

### 2.10. Enzyme-linked immunosorbent assay (ELISA)

Human IL-1*β* ELISA kits (Elabscience, E-EL-H0149c) were used to detect the concentration of IL-1*β* in NPCs under different treatment conditions. IL-1*β* antibody was added to the ELISA well, and standards and samples were added to the microplates. Each standard and sample were measured through ELISA at a wavelength of 450 nm. IL-1*β* concentration was evaluated according to the absorbance value.

### 2.11. RNA fluorescence *in situ* hybridization, RNA immunoprecipitation (RIP), and biotin-labeled miRNA capture

The subcellular localization of circ_0040039 and circ_0004354 in NPCs were measured using fluorescence *in situ* hybridization (FISH) assay. The Cy3-labeled specific circ_0040039 probe (Cy3-5'-CGGATGAACTTGACTAGAGAGACTT-3'- Cy3) and FITC-labeled specific circ_0004354 probe (FITC-5'- CAGAATGAACCAA CCTAGAGAGACT-3'-FITC) were designed and synthesized by Guangzhou Geneseed Biotech Co. (Guangzhou, China). These probes were diluted and denatured and then were placed in the culture hole to immerse the NPCs slide at 37°C overnight. Next, the NPCs slide were stained with 4,6-diamidino-2-phenylindole (DAPI) and mounted using rubber cement. The confocal images were captured using a confocal laser scanning microscope (TCS SP2 AOBS).

For RIP, a Magna RIP RNA-Binding Protein Immunoprecipitation Kit (Geneseed, Cat.No. P0101, China) was used to conduct RIP experiments. More specifically, 1 × 10^7^ NPCs were collected and resuspended in 1000 *μ*L buffer A containing 1% volume protease inhibitor and RNase inhibitors. The cell lysis supernatant (450 *μ*L) was incubated with 5 *μ*g AGO2 or control IgG antibody and protein A + G magnetic beads at 4°C overnight with rotation. Subsequently, the magnetic beads complex was cleaned six times in 1 mL buffer B. The isolated immunoprecipitated RNA was quantified by qRT-PCR to assess the relative expression of circ_0040039, circ_0004354, and miR-345-3p. Supplementary Table 3 shows the anti-AGO2 and IgG antibodies used in this study.

For miRNA pulldown assay, biotinylated miR-345-3p mimic or mimic NC (Guangzhou, China) were transfected into NPCs using Lipofectamine 8000 (Beyotime, China). Subsequently, M-280 Streptavidin Magnetic Beads (Invitrogen, USA) were incubated with the NPCs lysates at 4°C overnight. The biotin-coupled RNA complex bound to the beads was pulled down, and the relative expression of circ_0040039 and circ_0004354 in bound fractions was detected by qRT-PCR.

### 2.12. Dual-luciferase reporter assays

Targetscan database was employed to analyze the potential binding sites of miR-345-3p with circ_0040039 and circ_0004354 as well as FAF1 and TP73 mRNA 3'-UTR. Luciferase reporter vectors: psiCHECK2-Firefly luciferase -Renilla luciferase containing circ_0040039-765 bp or circ_0004354-197 bp or FAF1-500 bp or TP73 -500 bp wild type (WT) sequences or corresponding mutant (MUT) sequences, were constructed by Geneseed Biotech Co, respectively (Guangzhou, China). Human embryonic kidney (HEK) 293 T cells were added to 24-well plates at a density of 1 × 10^5^ cells per well. Subsequently, 1 *μ*g vectors and 100 *μ*L miR-345-3p mimic or miR-345-3p inhibitor or corresponding NC were co-transfected to HEK-293 T cells through 2 *μ*L lipofectamine 8000 (Beyotime, China). The luciferase activity was detected through the Luciferase Assay Kit (Promega, Madison, WI, USA) after transfection. The reporter genes' activation degree was calculated between different samples according to the obtained ratio of the relative light unit (RLU) value detected by Renilla luciferase is divided by the RLU value detected by firefly luciferase.

### 2.13. Statistical analysis

The data were analyzed and output as Figures by GraphPad Prism software 6 version. The statistical significance between the two groups was compared by an unpaired Student's *t-*test, whereas the differences among more than two groups were assessed by one-way analysis of variance followed by Turkey's multiple comparisons test. We carried out at least three independent experiments. Results are presented as mean ± standard deviation. *P*-value <0.05 was considered to be statistically significant.

## 3. Results

### 3.1. Identification of key circRNAs, miRNA, and mRNAs in IVDD

**Figure 1:**
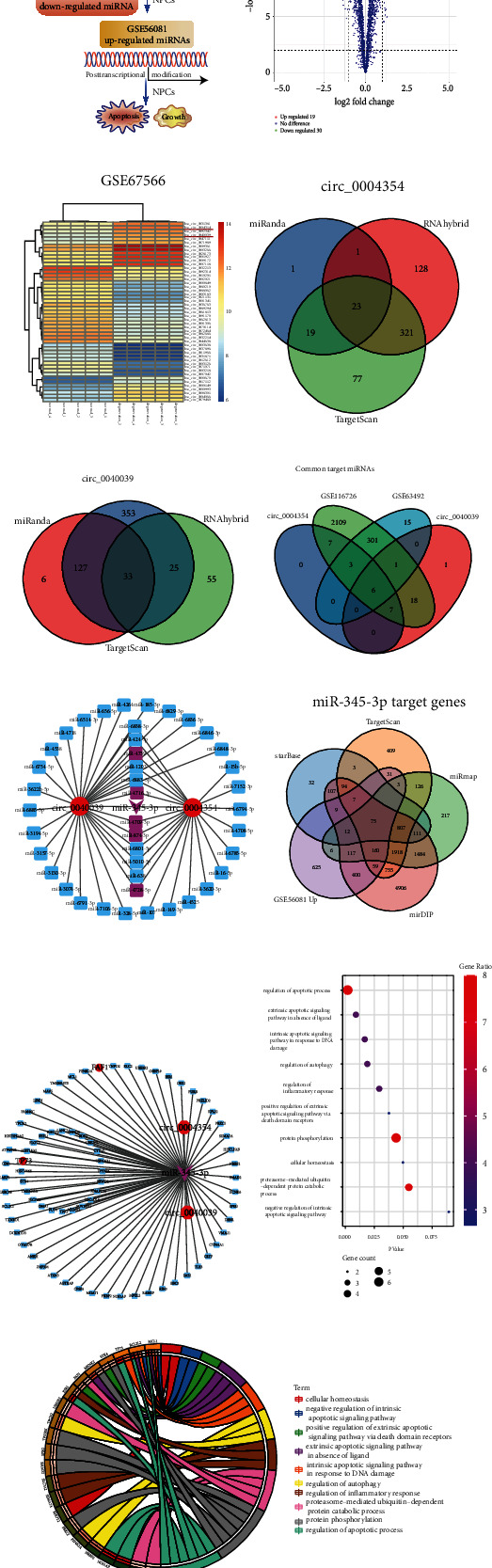
Prediction of key circRNAs, miRNA, and mRNAs in IVDD by bioinformatics analysis. (a) The flow chart delineates the steps for identifying and validating the biological functions of key RNAs in NP tissues and cells. (b) The volcano plot exhibited 49 DECs. Green points represent downregulated circRNAs, while red points represent upregulated circRNAs, circ_0040039 and circ_0004354 are indicated. (c) The clustering heat map showed the 49 DECs in IVDD, with rows indicating DECs and columns indicating tissues. The color scale varies from red to blue. Red, upregulation; blue, downregulation. Venn diagrams were employed to select the overlapping downstream miRNAs of circ_0004354 (d) and circ_0040039 (e) by the intersection of TargetScan, miRanda, and RNAhybrid databases. (f) Venn diagram displayed the common target miRNAs of circ_0040039 and circ_0004354, as predicted by different algorithms. (g) The circ_0040039/circ_0004354-miRNAs network was constructed based on the above prediction results. Circ_0040039/circ_0004354, IVDD-related common miRNAs, and miR-345-3p were indicated by a red ellipse, purple squares, and purple triangles, respectively. (h) 75 overlapping targets mRNAs of miR-345-3p were predicted by intersecting four different databases, and GSE56081 upregulated mRNAs. (i) Cytoscape software was utilized to construct the circ_0040039/circ_0004354 -miR-345-3p-mRNA interaction network, of which FAF1 and TP73 were indicated by a red ellipse. Bubble diagram (j) and Go chord diagram (k) revealed the predominant biological process of miR-345-3p is involved in.

Bioinformatics analysis and NPCs functional experiments were conducted to identify IVDD-related circRNAs, miRNA, and mRNAs (Figure 1(a)). The volcano plots (Figure 1(b)) and hierarchical clustering (Figure 1(c)) identified 49 differentially expressed circRNAs (DECs) based on the |log_2_ fold-change (FC)| > log_2_5 and -log_10_ false discovery rate (FDR) >2.  Table 2 lists the top five upregulated and downregulated DECs, of which circ_0040039 and circ_0004354 were the most remarkably upregulated circRNAs (Figure 1(b)). Furthermore, our previous study found that circ_0040039 remarkably promoted NPCs apoptosis and repressed NPCs growth [35]. These results predicted that circ_0040039 and circ_0004354 might be the key circRNAs in IVDD.

The Venn diagrams showed six common downstream miRNAs between the ones predicted by miRanda [26], RNAhybrid [27], TargetScan [28] databases and GSE63492/GSE116726 datasets to analyze the ceRNA mechanism of circ_0004354 and circ_0040039 (Figures 1(d)–1(f)). Also, miR-4728-5p, miR-4716-3p, miR-345-3p, and miR-874-3p were predicted to be downregulated (Figure 1(g) and Supplementary Figure 2(a)). Second, miR-345-3p expression was decreased, while miR-874-3p expression was increased in circ_0040039 and circ_0004354 overexpressing NPCs (Supplementary Figure 2(b)). Third, a miR-345-3p mimic significantly repressed NPCs death (Supplementary Figure 2(c)). Fourth, circ_0004354 and circ_0040039 were predicted to be the upstream circRNAs of miR-345-3p (Supplementary Figure 2(d)). Fifth, the miRanda [26] database predicted that circ_0004354 and circ_0040039 had two complementary sequences to the miR-345-3p seed region, whereas they had only one binding site to miR-874-3p (data not shown). Therefore, miR-345-3p was selected as a critical miRNA in IVDD.

Next, we predicted and analyzed the target mRNAs of miR-345-3p. A total of 75 overlapping IVDD-related mRNAs, encompassing FAF1 and TP73, were predicated by intersecting different algorithms, including Targetscan [28], starBase [29], miRmap [30], and mirDIP [31] databases and upregulated mRNAs of GSE56081 (Figure 1(h)); these were visualized using the Cytoscape software (Figure 1(i)). TP73, a TP53-related gene, was demonstrated to not only induce IL-1*β* expression [37] but also trigger cell cycle arrest and mitochondrial-dependent apoptosis by inducing cell cycle inhibitor cyclin-dependent kinase inhibitor 1A (CDKN1A/P21) and B-cell lymphoma 2 associated X protein (BAX) expression in a TP53-like manner [38–40]. Fas-associated factor 1 (FAF1), a Fas-binding protein, has been shown to initiate extrinsic apoptotic pathways by inducing CASP3 activation [41, 42]. The Bubble diagram (Figure 1(j)) and Go chord diagram (Figure 1(k)) indicated that miR-345-3p was probably involved in regulating FAF1-mediated extrinsic and TP73-mediated intrinsic apoptotic signaling pathway and inflammatory response. Moreover, TP73-mediated biological processes were mostly associated with the positive regulation of the permeability of the mitochondrial membrane, which involved the apoptotic process (Supplementary Figure 2(e)). Thus, these results predicted that circ_0040039/circ_0004354-miR-345-3p-FAF1/TP73 signaling network might be involved in the regulation of NPCs apoptosis and inflammatory response.

### 3.2. Verification of the levels of circ_0040039, circ_0004354, miR-345-3p, FAF1, and TP73 in NP tissues and TNF-*α*-treated NPCs

**Figure 2:**
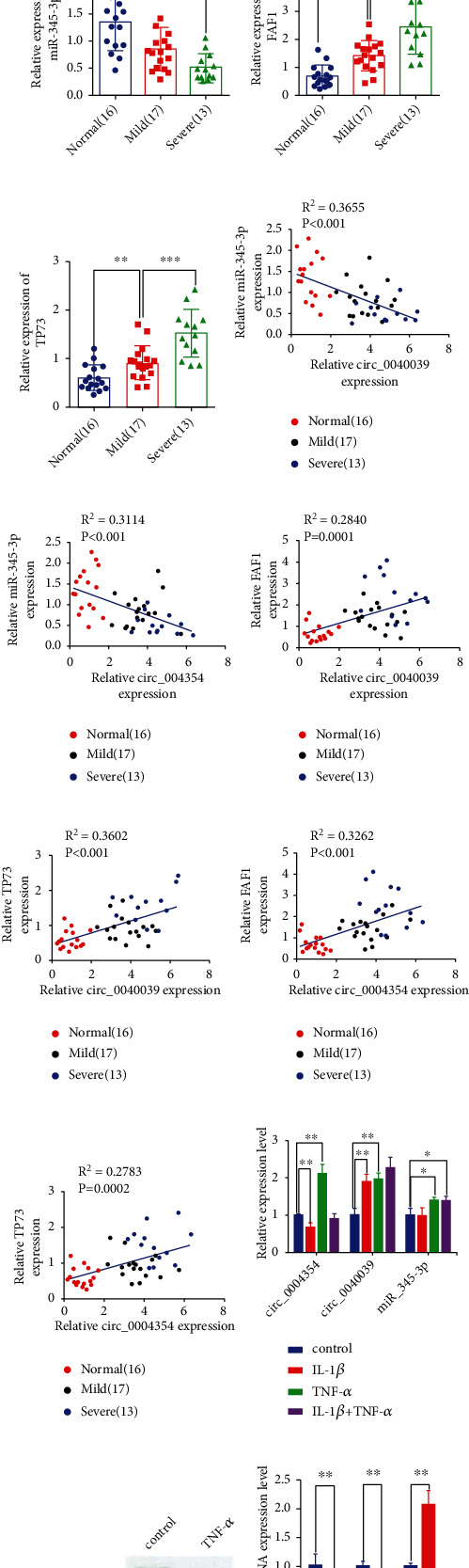
Verification of the levels of circ_0040039, circ_0004354, miR-345-3p, FAF1, and TP73 in NP tissues and TNF-*α*-treated NPCs. qRT-PCR experiment was utilized to measure the expression levels of circ_0040039 (a), circ_0004354 (b), miR-345-3p (c), FAF1 (d), and TP73 (e) in 16 human normal, 17 mild degenerative, and 13 severe degenerative NP tissues. ∗*P* <0.05, ∗∗*P* <0.01, ∗∗∗*P* <0.001. Spearman's rank correlation analysis was utilized to evaluate the correlation between circ_0040039 and miR-345-3p (f), circ_0004354 and miR-345-3p (g), circ_0040039 and FAF1 (h), circ_0040039 and TP73 (i), circ_0004354 and FAF1 (j), circ_0004354 and TP73 (k). (l) The expression levels of circ_0040039, circ_0004354 and miR-345-3p in NPCs were measured by qRT-PCR after treatment with TNF-*α*, IL-1*β* or both (TNF-*α* and IL-1*β*) (10 ng/ml). ∗*P* <0.05, ∗∗*P* <0.01. (M-N) TNF-*α* repressed ACAN and COL2 but promoted IL-1*β* mRNA and protein expression in NPCs. ∗∗*P* <0.01.

Consistent with the predicted results, circ_0040039, circ_0004354, FAF1, and TP73 were found to be remarkably upregulated, whereas miR-345-3p was downregulated in 30 degenerative NP tissues (Figures 2(a)–2(e)). Additionally, their expression was correlated with the severity of IVDD (Figures 2(a)–2E). Moreover, Pearson's correlation analysis revaled a negative correlation between circ_0040039/circ_0004354 and miR-345-3p expression and a positive correlation between circ_0040039/circ_0004354 and FAF1/TP73 expression, implying that circ_0040039 and circ_0004354 positively regulated FAF1/TP73 expression by co-adsorbing miR-345-3p in NP tissues (Figures 2(f)–2(k)).

Next, we utilized TNF-*α* and IL-1*β* to simulate the micro-environment of IVDD and find high-affinity pro-inflammatory factors to treat NPCs. Circ_0040039 and circ_0004354 expressions were found to be upregulated in response to TNF-*α* treatment (Figure 2(l)), which was consistent with our previous study [35]. In addition, the expression of miR-345-3p were also upregulated in response to TNF-*α* treatment (Figure 2(l)). The expression of miR-345-3p was elevated probably due to the fact that TNF-*α* transiently induced miR-345-3p expression to act as a buffer to alleviate IVDD. Additionally, TNF-*α* not only repressed ACAN and COL2 but also promoted IL-1*β* mRNA and protein expression in NPCs (Figures 2(m)–2(n)). Altogether, TNF-*α* elevated the key circRNAs, miRNA, and mRNAs expression and induced IVDD.

### 3.3. The biological functions of miR-345-3p in NPCs

**Figure 3:**
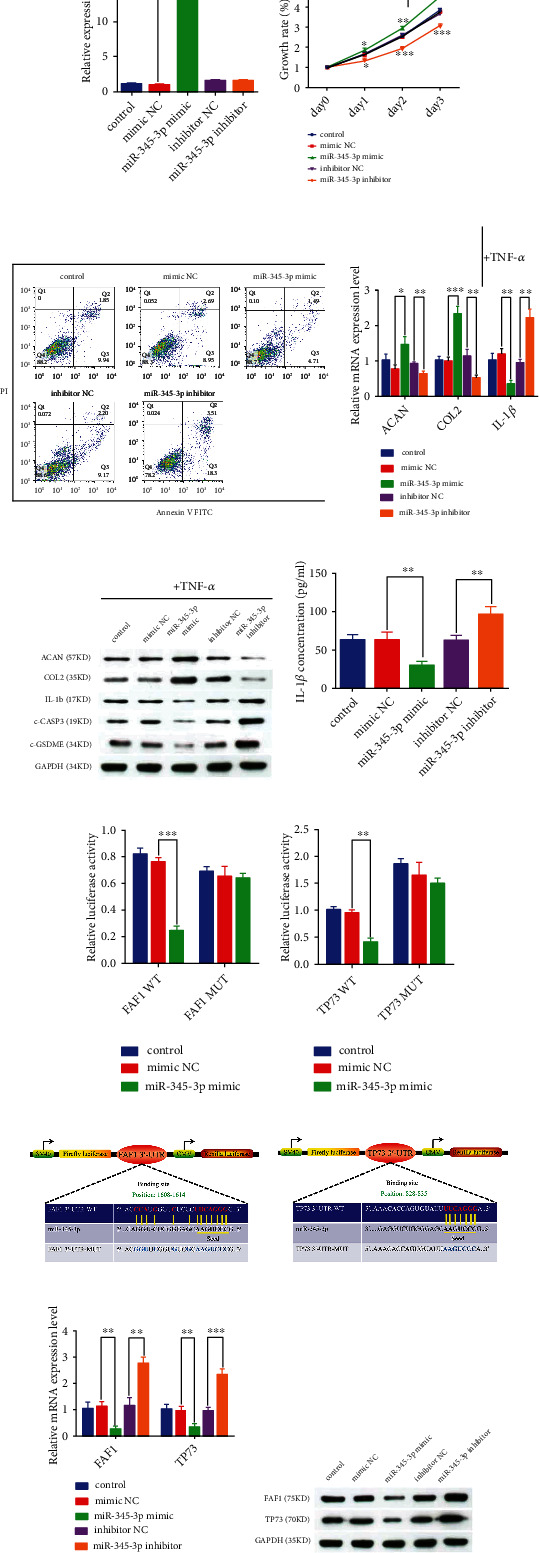
Identification of miR-345-3p biological functions and target genes. (A,C,F,K,L) NPCs were transfected with miR-345-3p mimic or miR-345-3p inhibitor or corresponding NC. (B,D,E) NPCs were treated with TNF-*α* after transfected with miR-345-3p mimic or miR-345-3p inhibitor or corresponding NC. (A) The expression levels of miR-345-3p were measured using qRT-PCR in NPCs. ∗∗∗*P* <0.001. (B) The NPCs growth rate was measured at the indicated time points by CCK-8 assay. ∗*P* <0.05, ∗∗*P* <0.01, ∗∗∗*P* <0.001. (C) NPCs apoptosis was measured by using a flow cytometry detection assay. Representative dot plots of apoptosis were displayed after Annexin V FITC/PI dual staining. (D) qRT-PCR assay corroborated that miR-345-3p promotes ACAN and COL2 but represses IL-1*β* mRNA expression levels in NPCs. ∗*P* <0.05, ∗∗*P* <0.01, ∗∗∗*P* <0.001. (E) Western blot analysis of the expression of ACAN, COL2, IL-1*β*, c-GSDME, and c-CASP3. (F) The concentration of IL-1*β* was detected by ELISA in NPCs. ∗∗*P* <0.01, ∗∗∗*P* <0.001. Schematic illustration of the putative binding sites of FAF1 (I) and TP73 (J) on miR-345-3p and the WT and MUT vector sequences of these mRNAs. Relative luciferase activity of FAF1 (G) and TP73 (H) were measured in the HEK-293 T cells after co-transfected WT or MUT FAF1/TP73 vectors with miR-345-3p mimic or mimic NC. ∗∗*P* <0.01, ∗∗∗*P* <0.001. (K) The mRNA expression levels of FAF1 and TP73 were measured in NPCs using the qRT-PCR assay. ∗∗*P* <0.01, ∗∗∗*P* <0.001. (L) Western blot assay confirmed that miR-345-3p repressed FAF1 and TP73 protein levels in NPCs.

Since circRNAs are known to exert their role in an miRNA-dependent manner; thus, we first studied the biological functions of miR-345-3p in NPCs. The miR-345-3p mimic remarkably upregulated miR-345-3p expression, whereas its expression was not altered by a miR-345-3p inhibitor (Figure 3(a)). NPCs growth inhibition (Figure 3(b)), death (Figure 3(c)), IL-1*β* expression (Figures 3(d)–3(f)), and ECM degradation (Figures 3(d)–3(e)) were decreased by the overexpression and enhanced by the knockdown of miR-345-3p in NPCs. Next, we wanted to test if miR-345-3p inhibited NPCs death and IL-1*β* production by regulating NPCs' PAoptosis. Thus, we monitored the expression of pro-apoptotic c-CASP3 and pro-pyroptotic c-GSDME. The miR-345-3p overexpressing NPCs confirmed that c-CASP3 and c-GSDME were decreased while miR-345-3p inhibitor exerted an opposite effect (Figure 3(e)). Thus, the gain-of-function and loss-of-function experiments revealed that miR-345-3p negatively regulated NPCs PAoptosis and inflammatory response in a CASP3 and GSDME-dependent manner.

### 3.4. MiR-345-3p repressed NPCs PAoptosis by directly inhibiting FAF1 and TP73

The potential binding sites of miR-345-3p with FAF1 and TP73 mRNA 3'-UTR were analyzed using the targetscan database to further determine how miR-345-3p played a protective role in NPCs (Figures 3(i)–3(j)). The dual-luciferase reporter assays revealed that the miR-345-3p mimic significantly suppressed FAF1 or TP73 wild type (WT) luciferase activity. In contrast, co-transfected FAF1 or TP73 mutant (MUT) vectors and miR-345-3p mimic or mimic NC group did not show any significant alteration (Figures 3(g)–3(h)). Second, miR-345-3p negatively regulated the gene and protein expression of FAF1 and TP73 in NPCs (Figures 3(k)–3(l)), which further supported that miR-345-3p could bind to FAF1 and TP73. These data suggested that FAF1 and TP73 were the direct targets of miR-345-3p.

**Figure 4:**
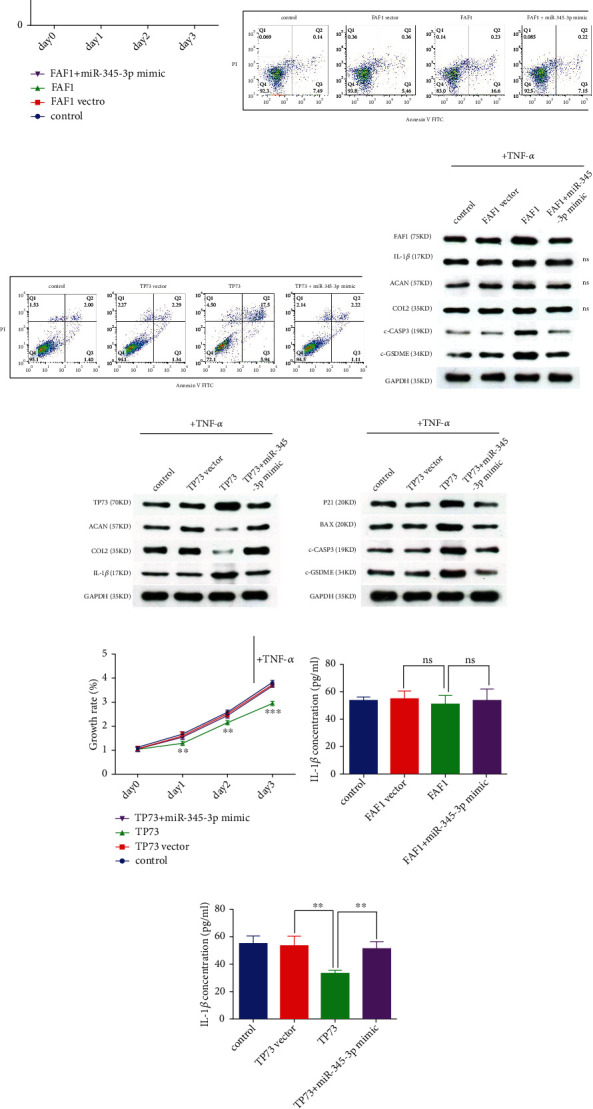
MiR-345-3p repressed NPCs degeneration by targeting FAF1 and TP73. (B,C,H,I) NPCs were transfected with FAF1/TP73 or empty vector or co-transfected with FAF1/TP73 and miR-345-3p mimic. (A,D,E,F,G) NPCs were treated with TNF-*α* after transfected with FAF1/TP73 or empty vector or co-transfected with FAF1/TP73 and miR-345-3p mimic. (A, G) The NPCs growth rate was detected at different time points using the CCK-8 assay. ∗*P* <0.05, ∗∗*P* <0.01, ∗∗∗*P* 0.001. (B, C) NPCs apoptosis was measured with Annexin V FITC/PI double staining by using a flow cytometry detection assay. (D) The protein levels of FAF1, ACAN, COL2, IL-1*β*, c-GSDME, and c-CASP3 were detected by Western blot assay. Western blot analysis of TP73, ACAN, COL2, and IL-1*β* (E) as well as P21, BAX, c-GSDME, and c-CASP3 (F) proteins levels. (G, H) The concentration of IL-1*β* was detected by ELISA in NPCs. ∗∗*P* <0.01.

FAF1 and TP73 overexpression vector significantly promoted the mRNA and protein expressions of FAF1 and TP73 in NPCs, respectively (Supplementary Figures 3(a)-3(d)). FAF1 overexpressing NPCs showed enhanced NPCs growth inhibition (Figure 4(a)), death (Figure 4(b)), and c-CASP3 and c-GSDME expression (Figure 4(d)), but had no influence on ECM and IL-1*β* expressions (Figures 4(d) and 4(h)), whereas miR-345-3p mimic could reverse the impact of FAF1. Furthermore, FAF1 was found to repress nuclear factor-*κ*B (NF-*κ*B) signaling pathway [42, 43], which is known to drive pro-apoptotic signaling [44, 45]. We speculated that the FAF1-CASP3- GSDME pathway induced upregulation of IL-1*β* expression might be offset by the decrease in IL-1*β* production caused by FAF1- NF-*κ*B pathway. However, we could not rule out the possibility that FAF1- NF-*κ*B pathway might be involved in the regulation of NPCs death.

TP73, which also promoted NPCs death (Figure 4(c)) and growth inhibition (Figure 4(g)), facilitated IL-1*β*, P21, BAX, c-CASP3, and c-GSDME expressions, and decreased ECM expressions (Figures 4(e)–4(f), and 4(i)), while miR-345-3p mimic reversed these effects. Thus, these results showed that miR-345-3p not only repressed NPCs PAoptosis by inhibiting FAF1-induced extrinsic apoptotic pathway and TP73-induced intrinsic mitochondrial apoptotic pathway as well as FAF1/TP73 mediated the activation of CASP3-GSDME pathway but also promoted NPCs growth, ECM production, and decreased IL-1*β* secretion by targeting TP73 and its downstream target genes.

### 3.5. The functional difference between circ_0040039 and circ_0004354 in NPCs in response to TNF-*α* treatments

**Figure 5:**
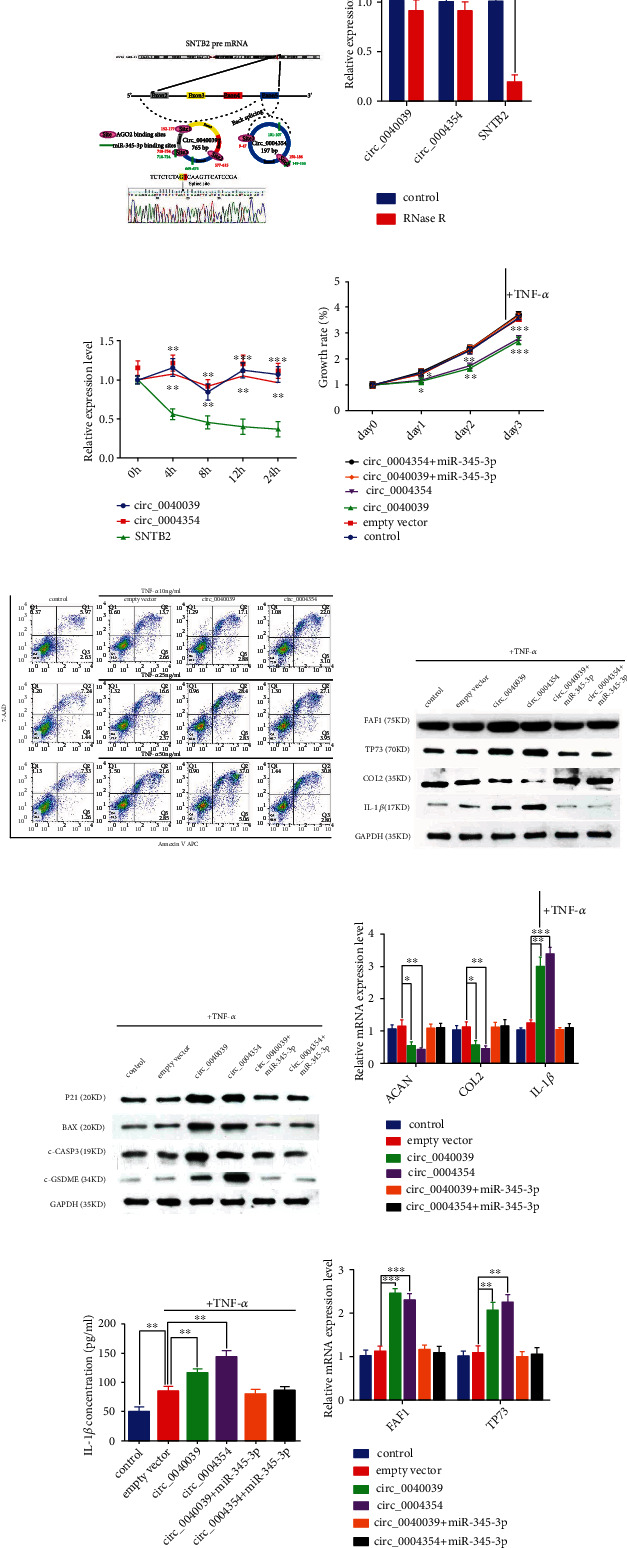
The functional difference between circ_0040039 and circ_0004354 in NPCs in response to TNF-*α* treatments. (A) The schematic illustration displays the basic characterization of circ_0040039 and circ_0004354. The red arrow represents the back-splicing site of circ_0040039. (B) Relative expression levels of circ_0040039, circ_0004354, and host gene *SNTB2* in NPCs were measured using qRT-PCR after treatment in the presence or absence of RNase R for 2 h. ∗∗∗*P* <0.001. (C) qRT-PCR was employed to measure the circ_0040039, circ_0004354, and *SNTB2* expression levels after treatment with ACT-D at different time points in NPCs. ∗∗*P* <0.01, ∗∗∗*P* <0.001. (D, F-I) NPCs were treated with TNF-*α* after transfected with circ_0040039/circ_0004354 or empty vector or co-transfected with circ_0040039/circ_0004354 and miR-345-3p mimic. (D)The NPCs growth rate was detected at different time points using the CCK-8 assay. ∗*P* <0.05, ∗∗*P* <0.01, ∗∗∗*P <0.001.* (E) NPCs apoptosis was measured with Annexin V APC/7-AAD double staining by using a flow cytometry detection assay in response to different concentrations of TNF-*α* treatments. Western blot analysis of FAF1, TP73, COL2, and IL-1*β* (F) as well as P21, BAX, c-GSDME, and c-CASP3 (G) proteins levels. (H) qRT-PCR measured the mRNA expression levels of ACAN, COL2, and IL-1*β* in NPCs. ∗*P* <0.05, ∗∗*P* <0.01, ∗∗∗p <0.001. (I) The concentration of IL-1*β* was detected using ELISA in NPCs. ∗*P* <0.05, ∗∗*P* <0.01. (J) TP73 and FAF1 mRNA expression levels were measured using qRT-PCR in NPCs. ∗∗*P* <0.01, ∗∗∗*P* <0.001.

Circ_0040039 is derived from the exons 2 to 5 of host gene *Sntb2* (chr16: 69279504-69318147), and circ_0004354 is derived from the exon 5 of *Sntb2* (chr16: 69317950-69318147) (Figure 5(a)). The length of the mature sequences from circ_0040039 and circ_0004354 were 765 bp and 197 bp, respectively. According to CircInteractome online database, circ_0040039 has three, and circ_0004354 has two binding sites with AGO2, of which each has one region overlapped with the seed sequences of miR-345-3p (Figure 5(a)). Next, we extracted circ_0040039, circ_0004354, and linear *Sntb2* mRNA from NPCs and treated them with or without RNase R to verify that the circ_0040039 and circ_0004354 were indeed circular. As expected, the expression of circ_0040039 and circ_0004354 was insignificantly modified in the presence of RNase R, whereas *Sntb2* expression was significantly decreased (Figure 5(b)). Also, circ_0040039 and circ_0004354 were more stable than *Sntb2* mRNA under the treatment of actinomycin D (ACT-d) because ACT-d could repress RNA synthesis (Figure 5(c)).

Previously, our study suggested that circ_0040039 and circ_0004354 had different biological characteristics [35]. Next, we constructed the overexpression vector of circ_0040039 and circ_0004354 to investigate their different biological functions and observed that their expression was increased approximately eight-fold in the over-expressed NPCs, respectively (Supplementary Figures 3(e)-3(f)). TNF-*α* is known to induce apoptosis [2, 3, 10, 11] and pyroptosis [11, 14]. Subsequently, we aimed to confirm whether TNF-*α* could induce circRNA-mediated NPCs PAoptosis. We used different concentrations of TNF-*α* to treat NPCs. When the TNF-*α* concentration was 10 ng/mL, circ_0004354 significantly promoted, whereas circ_0040039 slightly promoted NPCs death. With an increase in the concentration of TNF-*α*, the degree of NPC death increased rapidly in circ_0040039 but slowly in circ_0004354 over-expressed NPCs (Figure 5(e)). Additionally, the ability of circ_0040039 to inhibit NPCs growth was lesser than that of circ_0004354 (Figure 5(d)). Under the stimulation of TNF-*α*, the overexpression of circ_0040039 displayed the robust cleavage of apoptotic CASP3 and BAX and the slight cleavage of pyroptotic GSDME, while the over-expression of circ_0004354 showed the opposite results (Figures 5(f) and 5(g)). The expression of IL-1*β* was also elevated, but the effect of circ_0004354 in promoting IL-1*β* elevation was superior to circ_0040039 (Figures 5(f) and 5(i)). We also observed that circ_0004354 more significantly repressed ECM expression than circ_0040039 (Figures 5(f) and 5(h)). Moreover, the mRNA and protein expression of miR-345-3p target genes, FAF1 and TP73, were upregulated to varying degrees by circ_0040039 and circ_0004354 (Figure 5(j)). These phenotypes could be reversed in the presence of miR-345-3p mimic. Thus, these data revealed that circ_0040039 and circ_0004354 promoted NPCs PAoptosis and inflammatory response probably by co-adsorbing miR-345-3p; circ_0040039 had a stronger ability to promote apoptosis, while circ_0004354 was more inclined to promote pyroptosis.

### 3.6. Circ_0004354 competes with circ_0040039 to adsorb miR-345-3p in NPCs at 10 ng/mL TNF-*α* concentration

**Figure 6:**
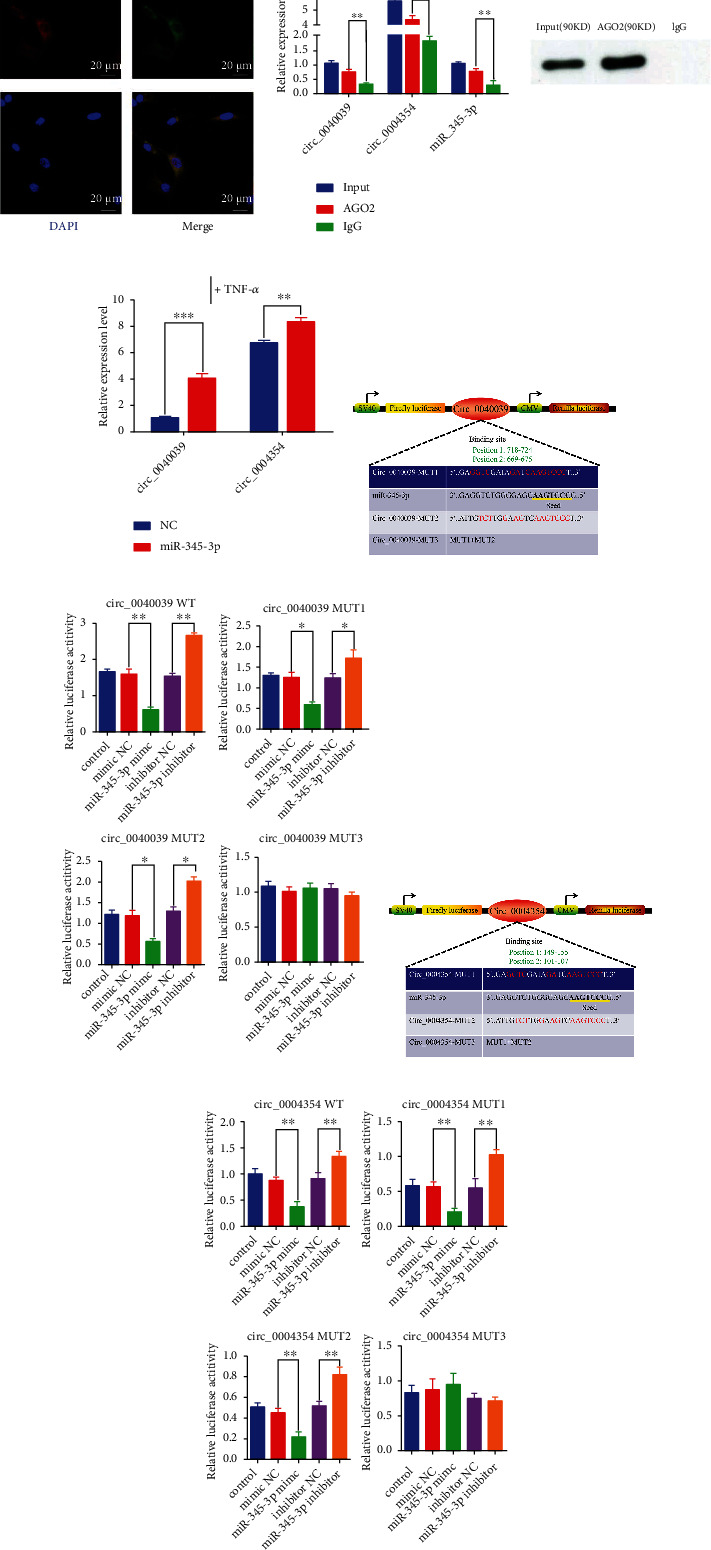
Circ_0040039 might compete with circ_0004354 to adsorb miR-345-3p in NPCs. (A-C) NPCs were treated with TNF-*α*. (A) Colocalization of circ_0040039 and circ_0004354 in the cytoplasm of NPCs was measured by FISH assay. Circ_0040039 and circ_0004354 were labeled with Cy3 (red) and FITC (green), respectively. Nuclei were stained with DAPI (blue). Scale bar =20 *μ*m. (B) The relationship between miR-345-3p and circ_0040039 and circ_0004354 was confirmed by the RIP experiment. Left, relative RNA levels compared with input. IgG acted as an NC. Right, the efficiency of AGO2 enrichment by anti-AGO2 was evaluated by western blot. ∗∗*P* <0.01, ∗∗∗*P* <0.001. (C) The levels of circ_0040039 and circ_0004354 in NPCs lysates captured by biotin-labeled miR-345-3p or NC probe. Relative RNA expression levels compared with NC. ∗∗*P* <0.01, ∗∗∗*P* <0.001. Schematic illustration of the putative binding sites of circ_0040039 (D) and circ_0004354 (F) on miR-345-3p and the WT and MUT vectors sequences of these circRNAs. (E, G) Luciferase reporter vectors carrying WT or MUT circ_0040039/circ_0004354 were co-transfected into HEK-293 T cells with miR-345-3p mimic or miR-345-3p inhibitor or corresponding NC. Relative luciferase activity was detected in the HEK-293 T cells. ∗*P* <0.05, ∗∗*P* <0.01, ∗∗∗*P* <0.001.

**Figure 7:**
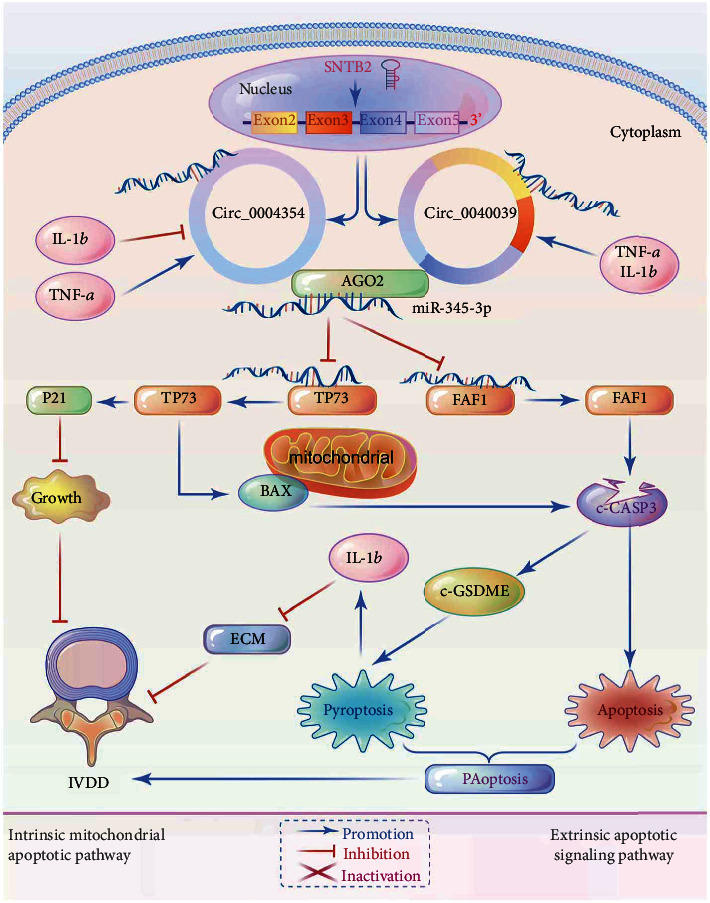
The schematic sketch of mechanisms by which circ_0004354 competes with circ_0040039 to induce IVDD.

Next, FISH, RIP, and RNA pulldown assays were conducted at a TNF-*α* concentration of 10 ng/mL to further elucidate the mechanism of functional difference between circ_0040039 and circ_0004354 in NPCs. This concentration was selected since circ_0040039 and circ_0004354 expressions were relatively low in normal NPCs and relatively high in 10 ng/mL TNF-*α*-induced NPCs. First, the FISH assay confirmed that circ_0040039 and circ_0004354 were co-localized and both preferentially cytoplasmic (Figure 6(a)), implying that there may be an interaction between them. Second, the RIP results revealed that circ_0040039, circ_0004354, and miR-345-3p were remarkably enriched by the anti-AGO2 antibody rather than the anti-IgG antibody, and the expression abundance of circ_0004354 was higher than circ_0040039, suggesting that they all existed in RISC (Figure 6(b)). Third, a biotinylated miR-345-3p pulldown assay showed that the circ_0004354 had a more remarkable enrichment compared with circ_0040039 in NPCs (Figure 6(c)). Next, a circ_0040039 or circ_0004354 fragment with WT or MUT complementary binding sites for miR-345-3p were established and inserted into psiCHECK2 luciferase reporter vectors, respectively, to further verify their interaction (Figures 6(d) and 6(f)). The results of the assay found that miR-345-3p mimic remarkably decreased and miR-345-3p inhibitor markedly elevated the luciferase activity of circ_0040039 or circ_0004354 WT and MUT1-2 reporters, whereas the luciferase activity of MUT3 reporters (sites 1 and 2 mutate together) was not noticeably altered, revealing that both circ_0040039 and circ_0004354 could directly bind to miR-345-3p via the two complementary target sites (Figures 6(e) and 6(g)). These results indicated that circ_0004354 might have a stronger binding ability to miR-345-3p at 10 ng/mL TNF-*α* concentration. The schematic sketch of mechanisms by which circ_0004354 competes with circ_0040039 to induce IVDD was showed in Figure 7.

## 4. Discussion

Previous studies have shown that the DECs play a critical role in the pathological process of IVDD by regulating NPCs' biological functions [7, 17–21]. However, the biological functions and mechanisms of multiple circRNAs in IVDD have not yet been identified. The present study identified circ_0040039 and circ_0004354, both of which originated from the circularization of *SNTB2* gene exons, triggering TNF-*α*-induced NPCs PAoptosis through the competitive adsorption of miR-345-3p. Our conclusions were based on the following observations: 1) In the presence of TNF-*α*, FAF1-activated CASP3-GSDME, resulting in extrinsic NPCs PAoptosis without an increase in IL-1*β* expression; while TP73 activated CASP3-GSDME pathway, resulting in intrinsic NPCs PAoptosis with an increase in IL-1*β* expression and a decrease in ECM components expression; 2) MiR-345-3p repressed NPCs PAoptosis, IL-1*β* expression and promoted the expression of ECM components by targeting FAF1 and TP73; 3) Both circ_0040039 and circ_0004354 promoted TNF-*α*-induced NPCs PAoptosis, of which circ_0040039 had a stronger ability to promote NPCs apoptosis, while circ_0004354 was more inclined to promote NPCs pyroptosis; 4) Circ_0004354 might compete with circ_0040039 to adsorb miR-345-3p in NPCs, and there was more circ_0004354 binding to miR-345-3p at TNF-*α* concentration of 10 ng/mL. Thus, this work unraveled a whole new mechanism of how circ_0040039 and circ_0004354 induced mixed inflammatory cell death, PAoptosis, by the competitive adsorption of miR-345-3p in NPCs.

Until now, several studies have reported that circRNAs function as ceRNA to modulate the progression of IVDD in a miRNAs-dependent manner, predominately encompassing ECM-related, apoptosis-related, and inflammation-related pathways [7, 17–21] Cheng et al. [18] corroborated that circ-VMA21 could alleviate pro-inflammatory cytokines-induced NPCs death and ECM decomposition by repressing the miR-200c-XIAP pathway both *in vitro* and *in vivo*. Wang et al. [21] showed that circ-4099 could promote ECM synthesis and repress the excretion of TNF-*α* and IL-1*β*, but the molecular mechanism involved in the dysregulation of IL-1*β* expression is still unclear. Our previous study demonstrated that circ_0040039 could promote NPCs apoptosis and repress NPCs growth [35]. Shen et al. [36] validated that circSERPINE2 could repress chondrocytes apoptosis and ECM synthesis by targeting miR-1271 and ETS-related gene. Our current study verified that circ_0004354 might compete with circ_0040039 to trigger TNF-*α*-induced NPCs PAoptosis, inflammatory response, growth inhibition, and ECM degradation by targeting the miR-345-3p-FAF1/TP73 axis. Nevertheless, we could not rule out the possibility that the increase in IL-1*β* levels in NPCs also might have enhanced NPC's PAoptosis and ECM degradation. Additionally, further studies are required to determine whether TP73 directly combined with ACAN and COL2 to inhibit their expression.

The cell death pathways have long been believed to function in parallel with little or no overlap. Recent studies have shown that apoptosis and pyroptosis are closely linked and could cross-regulate each other. Hou et al. [46] found that the macrophage-derived TNF-*α*-activated caspase8, in the presence of GSDMC activated by hypoxia, switching apoptosis to pyroptosis. GSDME was confirmed to switch CASP3-mediated apoptosis to pyroptosis [14, 15]. Karki et al. [11] demonstrated the synergism between TNF-*α* and IFN-*γ* triggering a mixed cell death PANoptosis composed of apoptosis, pyroptosis, and necroptosis. Here, we found a new circRNAs-mediated inflammatory cell death, PAoptosis, which is composed of apoptosis and pyroptosis in NPCs. Intriguingly, the degree of circ_0040039 promoting NPCs PAoptosis was positively correlated with the concentration of TNF-*α*, whereas the degree of circ_0004354 promoting NPCs PAoptosis gradually slowed down with an increase in TNF-*α* concentration. The possible reasons were as follows: First, the release of IL-1*β* increased with an increase in NPCs PAoptosis, IL-1*β* not only negatively feedback inhibited the expression of circ_0004354, which slowed down the rate of PAoptosis and release of IL-1*β*, but also induced circ_0040039 expression, which accelerated the rate of PAoptosis and the release of IL-1*β*, forming a feedback regulation mechanism. Furthermore, RIP and RNA pulldown assays showed that there was more circ_0004354 enrichment, suggesting circ_0004354 might have a stronger binding ability to miR-345-3p at 10 ng/mL TNF-*α* concentration. However, further studies are required to determine the binding ability of circ_0040039/circ_0004354 and miR-345-3p and the effects on the phenotype of NPCs under high concentration of TNF-*α* or high concentration of TNF-*α* + high concentration of IL-1*β*.

Therefore, we propose the following IVDD pathological mechanism model (Figure 7): *Sntb2* gene was cut into multiple circRNAs, such as circ_0040039 and circ_0004354 under certain pathological conditions. In the early stages of IVDD, the concentration of micro-environmental pro-inflammatory cytokines (such as TNF-*α*) was lower, so circ_0004354 might have a stronger binding ability to bind with miR-345-3p, which powerfully promotes TP73 and slightly promotes FAF1 expression, thereby predominantly inducing NPCs pyroptosis and releasing IL-1*β*. With the increased secretion of pro-inflammatory cytokines in the micro-environment, IL-1*β* negatively feedback inhibited the expression of circ_0004354, which slowed down the rate of PAoptosis and the secretion of pro-inflammatory cytokines and strived to balance the micro-environment homeostasis again. Simultaneously, the pro-inflammatory cytokines (such as TNF-*α* and IL-1*β*) might induce the expression of circ_0040039, which antagonized the binding of circ_0004354 to miR-345-3p and enhanced the ability of circ_0040039 to bind to miR-345-3p, inducing NPCs apoptosis and releasing IL-1*β*. Therefore, we hypothesized that under different inflammatory factor concentration gradients, circ_0040039/circ_0004354 and miR-345-3p had different binding abilities, forming a feedback regulation mechanism, continuously releasing inflammatory factors, and finally forming an inflammatory cascade, which initiated or aggravated IVDD.

Several circRNAs were reported to have similar functions with their linear counterparts [47, 48]. For example, a zinc finger with KRAB and SCAN domains 1 (*ZKSCAN1*) gene and its corresponding circRNA (circZKSCAN1) both repressed cell growth by mediating distinct signaling pathways [48]. In this study, we identified the role of SNTB2 related circRNAs (circ_0040039 and circ_0004354) on NPCs, while SNTB2 functions remained unclear. Notably, we found that miR-345-3p also repressed SNTB2 mRNA expression (data not shown), but its role in NPCs still unknown. The relationship between SNTB2 and SNTB2-derived circRNAs needs further investigation.

However, there were also several limitations to this study. First, the microarray data were obtained from GEO, and the clinical sample size was relatively small since the normal NP tissues were difficult to obtain in clinical practice. Second, the animal experiments will be performed in the future. Third, we have not silenced circRNAs to validate their functions, since their expression was relatively low in normal NPCs. Finally, further studies are required to examine the levels of IL-1*β* and PAoptosis after co-transfection of circ_0040039 and circ_0004354 to NPCs and rat models.

## 5. Conclusion

Here, we demonstrated that circ_0004354 might compete with circ_0040039 to adsorb miR-345-3p to positively regulate FAF1/TP73 and their downstream target genes in the induction of low concentration of TNF-*α*, thereby promoting NPCs PAoptosis, inflammatory response, growth inhibition, and ECM degradation. These findings offer a novel insight into the circRNAs-mediated the posttranscriptional regulatory network in IVDD, which would contribute to the understanding of the pathological mechanism of IVDD to develop an invaluable therapeutic approach to IVDD diseases.

## Figures and Tables

**Table 1:** Basic information of the microarray dataset from GEO.

Data source.	Platform	Sample size(D/N)	Region	First author	Year	RNA type	Screening criteria
GSE67566	GPL19978	5/5	China	Lan pH	2016	circRNA	FC>5, FDR<0.01
GSE63492	GPL19449	5/5	China	Lan PH	2016	miRNA	
GSE116726	GPL20712	3/3	China	Ji ML	2018	miRNA	FC>2, FDR<0.05
GSE56081	GPL15314	3/3	China	Wan Zy	2014	mRNA	FC>2, FDR<0.05

Abbreviations: GEO Gene Expression Omnibus; D degeneration, N normal; circRNA, circular RNA; miRNA, microRNA; mRNA, messenger RNA; FC, fold change; FDR, false discovery rate.

**Table 2:** Top 5 up-regulated and down-regulated DECs.

CircRNA.	Expression	GeneSymbol	logFC	FDR
hsa_circ_0040039	Up	SNTB2	2.980678	0.00000000000000392
hsa_circ_0004354	Up	SNTB2	2.928536	0.000000000000000698
hsa_circ_0028173	Up	ATP2A2	2.924426	0.000000000000000698
hsa_circ_0007158	Up	FAM169A	2.862396	0.00000000000000172
hsa_circ_0092342	Up	RPL27A	2.839063	0.0000000000000387
hsa_circ_0036763	Down	SEMA4B	-3.300365	0.000000000000016
hsa_circ_0082686	Down	PARPI2	-3.255699	0.000000000000219
hsa_circ_0003258	Down	ZNF652	-3.175986	0.0000000000000192
hsa_circ_0072464	Down	ARL15	-3.057012	0.000000000000000698
hsa_circ_0003526	Down	SPG21	-3.048583	0.000000000000000806

## Data Availability

All data relevant to the study are included in the article or uploaded as supplementary information.

## Supplementary Materials

Supplementary 1 Supplementary Table 1. The detailed information of patients with different diseases and Pfirrmann grades.

Supplementary 2 Supplementary Table 2. All primers used in this study.

Supplementary 3 Supplementary Table 3. The list of antibodies used in this study.

Supplementary 4 Supplementary Figure 1. The partial data on the patients' MRI information. Patients with Pfirrmann grade I/II were assigned to the normal group, whereas those with Pfirrmann grade III/IV constituted mild degeneration and grade V constituted the severe degeneration group. Each group listed at least one typical MRI results. The patient's diagnosis and Pfirrmann grade were showed below the figures. The red arrow showed the levels of the diseased intervertebral disc.

Supplementary 5 Supplementary Figure 2 Validation of circ_0040039/circ_0004354 and miR-345-3p as key circRNAs and miRNA in NPCs. (A) IVDD-related common downstream miRNAs of circ_0040039 and circ_0004354 were displayed in the volcano plot based on the analysis of GSE116726. (B) The expression of the above miRNAs was detected by qRT-PCR after transfected with circ_0040039 or circ_0004354 in NPCs. ∗∗*P* <0.01. (C) Flow cytometry assay was used to evaluate NPCs apoptosis after transfection with miR-345-3p mimic. (D) The upstream circRNAs of miR-345-3p were predicted by different algorithms. (E) The TP73-mediated biological process was visualized by the clueGO plugin in Cytoscape software.

Supplementary 6 Supplementary Figure 3 Validation of the overexpression effect of circ_0040039, circ_0004354, FAF1, and TP73 in NPCs. The expression levels of FAF1 (A, C), TP73 (B, D), circ_0040039 (E), and circ_0004354 (F) were significantly increased in NPCs after transfected with the above overexpression vectors, respectively. ∗∗∗*P* <0.001.
