# Supplementary material for: Circ_0004354 might compete with circ_0040039 to induce NPCs death and inflammatory response by targeting miR-345-3p-FAF1/TP73 axis in intervertebral disc degeneration
Source: Oxid Med Cell Longev. 2022 Jan 7;2022:2776440. doi: 10.1155/2022/2776440 (PMC8760533; doi:10.1155/2022/2776440)
Supplement: Supplementary 1 — Supplementary Table 1. The detailed information of patients with different diseases and Pfirrmann grades. [file 2776440.f1.pdf]

Supplementary Table 1 The detailed data for each patient with different diseases

Number	Gender	Age	Level	Diagnosis	Pfirrmann
Nor-1	F	22	T12-L1	Thoracolumbar fracture	I
Nor-2	M	32	T12-L1	Thoracolumbar fracture	II
Nor-3	M	19	T12-L1	Thoracolumbar fracture	II
Nor-4	F	21	T12-L2	Thoracolumbar fracture	I
Nor-5	M	31	L1-L2	Thoracolumbar fracture	II
Nor-6	M	49	T12-L1	Spinal cord injury	II
Nor-7	M	28	T12-L1	Spinal cord injury	I
Nor-8	F	23	T12-L1	Scoliosis	I
Nor-9	F	12	L2-L3	Scoliosis	I
Nor-10	F	10	T12-L1	Scoliosis	I
Nor-11	M	41	L4-L5	Lumbar disc herniation(L5-S1)	II
Nor-12	F	37	L4-L5	Lumbar disc herniation(L5-S1)	II
Nor-13	F	24	L5-S1	Lumbar spondylolisthesis	I
Nor-14	M	18	L5-S1	Lumbar spondylolisthesis	I
Nor-15	M	29	L5-S1	Lumbar spondylolysis	I
Nor-16	M	32	L5-S1	Lumbar spondylolysis	II
Mild-1	M	57	L3-L4	Lumbar disc herniation	IV
Mild-2	M	57	L4-L5	Lumbar disc herniation	IV
Mild-3	F	31	L4-L5	Lumbar disc herniation	III
Mild-4	F	71	L3-L4	Lumbar disc herniation	IV
Mild-5	M	44	L4-L5	Lumbar disc herniation	III
Mild-6	M	55	L5-S1	Lumbar disc herniation	IV
Mild-7	M	67	L5-S1	Lumbar disc herniation	IV
Mild-8	F	20	L5-S1	Lumbar disc herniation	IV
Mild-9	M	33	L5-S1	Lumbar spondylolysis	III
Mild-10	M	40	L5-S1	Lumbar spondylolysis	IV
Mild-11	F	52	L4-L5	Lumbar spondylolysis	IV
Mild-12	M	64	L5-S1	Lumbar spondylolysis	IV
Mild-13	F	41	L4-L5	Lumbar spondylolisthesis	IV
Mild-14	F	32	L4-L5	Lumbar spondylolisthesis	III
Mild-15	M	65	L4-L5	Lumbar spondylolisthesis	IV
Mild-16	M	45	L5-S1	Lumbar spondylolisthesis	IV
Mild-17	F	31	L5-S1	Lumbar spondylolisthesis	III
Severe-1	M	51	L4-L5	Lumbar disc herniation	V
Severe-2	F	64	L4-L5	Lumbar disc herniation	V
Severe-3	F	70	L4-L5	Lumbar disc herniation	V
Severe-4	M	66	L5-S1	Lumbar disc herniation	V
Severe-5	F	74	L5-S1	Lumbar disc herniation	V
Severe-6	M	76	L4-L5	Lumbar disc herniation	V
Severe-7	F	63	L4-L5	Lumbar spondylolisthesis	V
Severe-8	M	54	L4-L5	Lumbar spondylolisthesis	V

Severe-9	M	57	L5-S1	Lumbar spondylolisthesis	V
Severe-10	F	64	L5-S1	Lumbar spondylolisthesis	V
Severe-11	F	60	L4-L5	Lumbar spinal stenosis	V
Severe-12	F	72	L5-S1	Lumbar spinal stenosis	V
Severe-13	M	68	L5-S1	Lumbar spinal stenosis	V

Abbreviations: Nor, Normal; Mild, Mild degeneration; Severe, Severe degeneration; F, Female; M, Male
