# Supplementary material for: Circ_0004354 might compete with circ_0040039 to induce NPCs death and inflammatory response by targeting miR-345-3p-FAF1/TP73 axis in intervertebral disc degeneration
Source: Oxid Med Cell Longev. 2022 Jan 7;2022:2776440. doi: 10.1155/2022/2776440 (PMC8760533; doi:10.1155/2022/2776440)
Supplement: Supplementary 2 — Supplementary Table 2. All primers used in this study. [file 2776440.f2.pdf]

Supplementary Table 2 The qRT-PCR primers used in this study.

Gene	Forward primer (5’-3’)	Reverse primer(5’-3’)
circ_0040039	CAAGGAAGTCTCTCTAGTCA	TGCACCTTCCCACGGCAGAT
circ_0004354	TTCAGGATATCGTTGGTTCATTCTGGCTC	TACCTCCGCGGCTAGAGAGACTTCC
FAF1	ACCAACGTGTTCTGCTCACA	AGTCCGAATGGTTTGTGCCA
TP73	CCTCCCAAGGGTTACAGAGC	CCAGATGGTCATGCGGTACT
ACAN	TGTAACCCAGGCTCCAAC	GCAGCCCACTTAGGTCC
COL2	GCCTGAAGGGACACCG	CCAGGGATTCCATTAGCAC
IL-1β	CGCCAGTGAAATGATGGCTTA	GTGGTGGTCGGAGATTCGTAG
GAPDH	AGAAGGCTGGGGCTCATTTG	GCAGGAGGCATTGCTGATGAT
U6	CTCGCTTCGGCAGCACA	AACGCTTCACGAATTTGCGT
miR-345-3p	GTCGTATCCAGTGCAGGGTCCGAGGT<br>ATTCGCACTGGATACGACCctccagac	ATGGTTCGTGGGgccctgaacgaggggtctgg
miR-345-3p	Com R: GTGCAGGGTCCGAGGT	
