# Supplementary material for: Circ_0004354 might compete with circ_0040039 to induce NPCs death and inflammatory response by targeting miR-345-3p-FAF1/TP73 axis in intervertebral disc degeneration
Source: Oxid Med Cell Longev. 2022 Jan 7;2022:2776440. doi: 10.1155/2022/2776440 (PMC8760533; doi:10.1155/2022/2776440)
Supplement: Supplementary 3 — Supplementary Table 3. The list of antibodies used in this study. [file 2776440.f3.pdf]

Supplementary Table 3 Antibody for western blotting and RIP

Antibody	Company	Cat No.	Dilution	Description
FAF1	Abcam	ab183045	1:50000	Rabbit monoclonal (EPR14754)
TP73	Abcam	ab215038	1:1000	Rabbit monoclonal (EPR19884)
ACAN	Abcam	ab3778	1:200	Mouse monoclonal (6-B-4)
COL2	Abcam	ab188570	1:5000	Rabbit monoclonal (EPR12268)
IL-1β	Abcam	ab9722	1:1000	Rabbit polyclonal
P21	Abcam	ab109520	1:2000	Rabbit monoclonal [EPR362]
BAX	Abcam	ab32503	1:2000	Rabbit monoclonal [E63]
CASP3	Abcam	ab32351	1:5000	Rabbit monoclonal (E87)
AGO2	Abcam	ab32381	1:1000	Rabbit polyclonal
IgG	Sino Biological	CR1	1:1000	Rabbit polyclonal
GSDME	Abcam	ab215191	1:1000	Rabbit monoclonal (EPR19859)
GAPDH	Proteintech	10494-1-ap	1:20000	Rabbit
β-actin	Proteintech	66009-1-1g	1:10000	Rabbit
