# Supplementary material for: Circ_0004354 might compete with circ_0040039 to induce NPCs death and inflammatory response by targeting miR-345-3p-FAF1/TP73 axis in intervertebral disc degeneration
Source: Oxid Med Cell Longev. 2022 Jan 7;2022:2776440. doi: 10.1155/2022/2776440 (PMC8760533; doi:10.1155/2022/2776440)
Supplement: Supplementary 4 — Supplementary Figure 1. The partial data on the patients' MRI information. Patients with Pfirrmann grade I/II were assigned to the normal group, whereas those with Pfirrmann grade III/IV constituted mild degeneration and grade V constituted the severe degeneration group. Each group listed at least one typical MRI results. The patient's diagnosis and Pfirrmann grade were showed below the figures. The red arrow showed the levels of the diseased intervertebral disc. [file 2776440.f4.pdf]

Normal | Normal | Mild | Mild | Mild | Severe

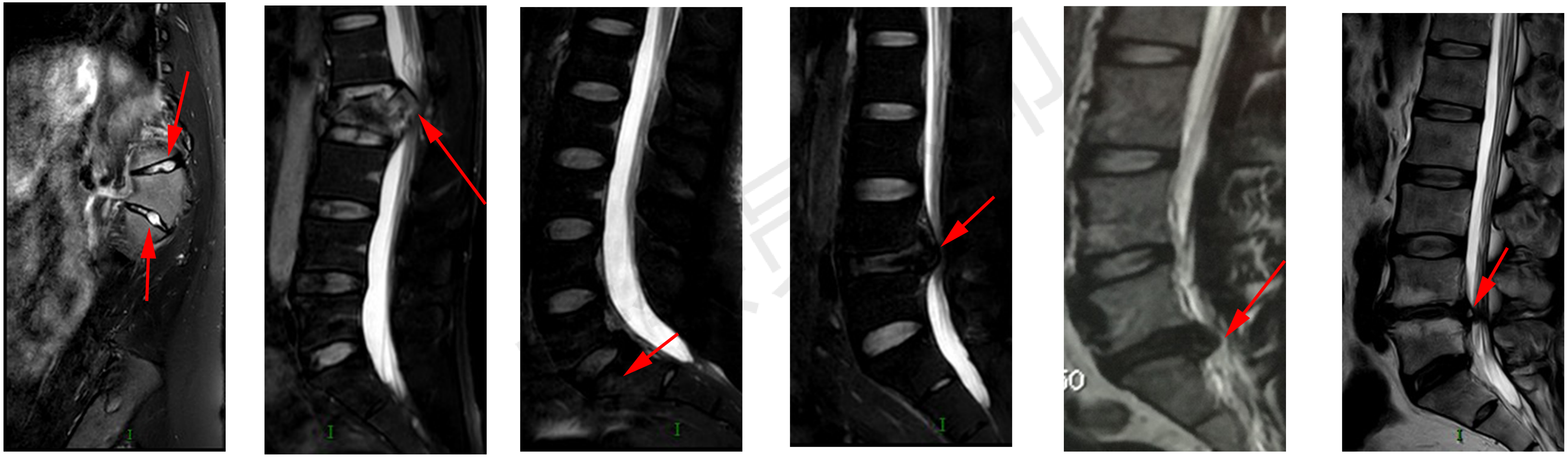

Scoliosis I | Thoracolumbar fracture II | Lumbar spondylolysis III | LDH III | LDH IV | LDH V
