# Supplementary figures and images for: Circ_0004354 might compete with circ_0040039 to induce NPCs death and inflammatory response by targeting miR-345-3p-FAF1/TP73 axis in intervertebral disc degeneration
Source: Oxid Med Cell Longev. 2022 Jan 7;2022:2776440. doi: 10.1155/2022/2776440 (PMC8760533; doi:10.1155/2022/2776440)

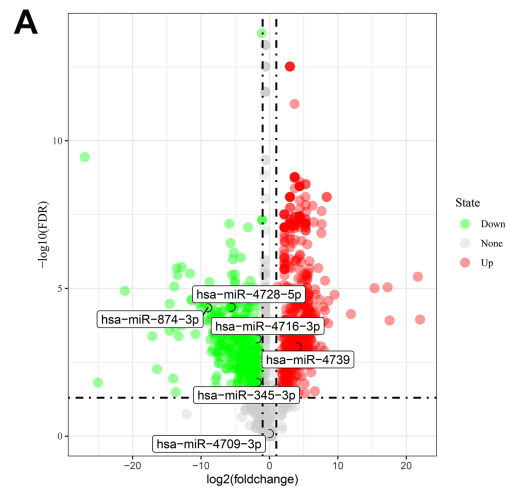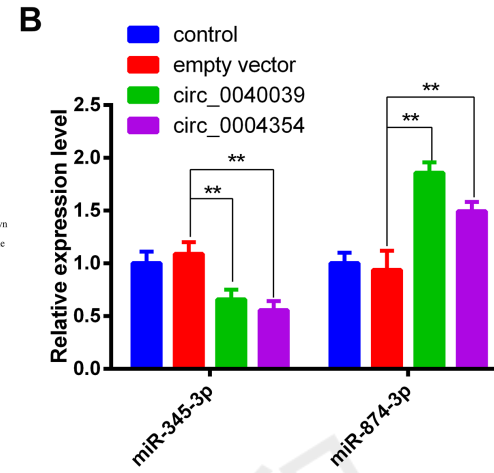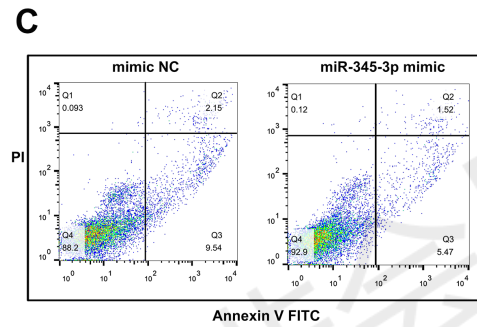

**D miR-345-3p upstream circRNA**

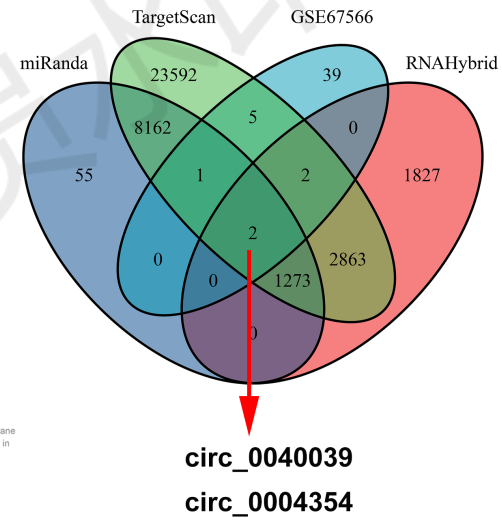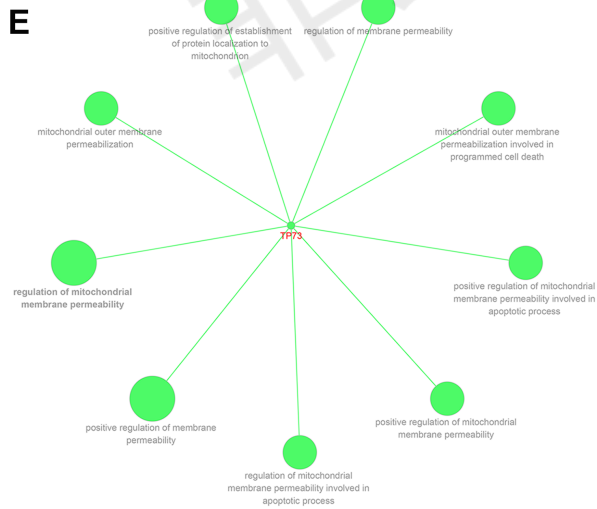

Supplement: Supplementary 5 — Supplementary Figure 2 Validation of circ_0040039/circ_0004354 and miR-345-3p as key circRNAs and miRNA in NPCs. (A) IVDD-related common downstream miRNAs of circ_0040039 and circ_0004354 were displayed in the volcano plot based on the analysis of GSE116726. (B) The expression of the above miRNAs was detected by qRT-PCR after transfected with circ_0040039 or circ_0004354 in NPCs. ∗∗P <0.01. (C) Flow cytometry assay was used to evaluate NPCs apoptosis after transfection with miR-345-3p mimic. (D) The upstream circRNAs of miR-345-3p were predicted by different algorithms. (E) The TP73-mediated biological process was visualized by the clueGO plugin in Cytoscape software. [file 2776440.f5.pdf]

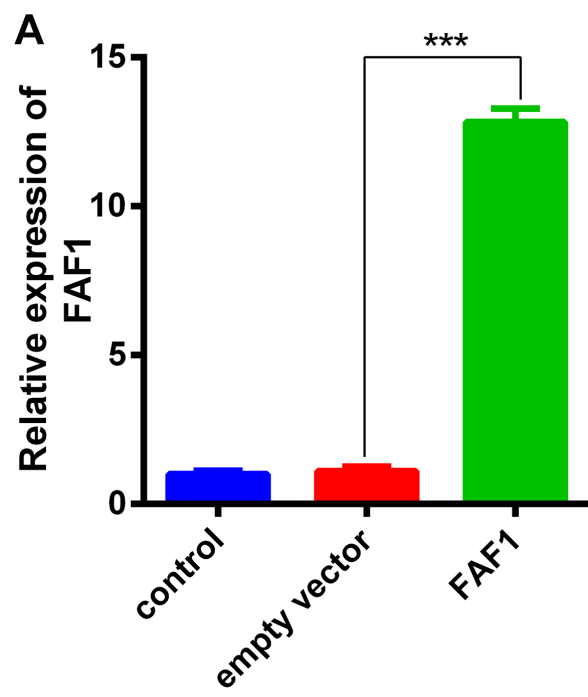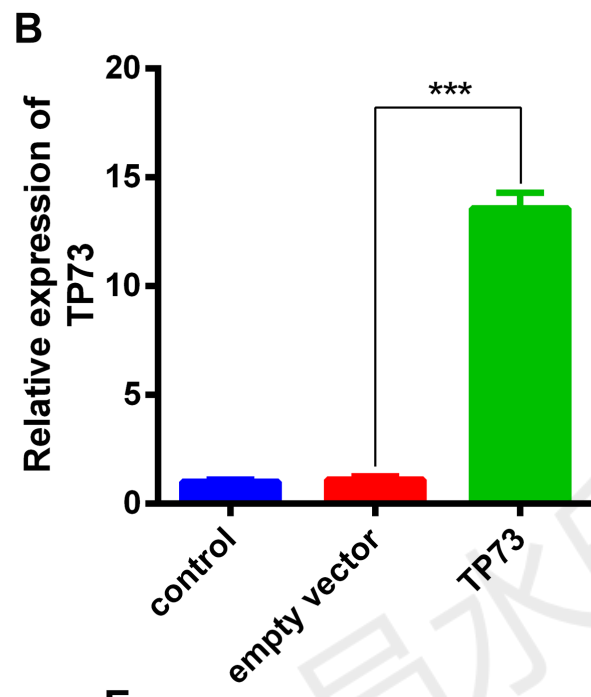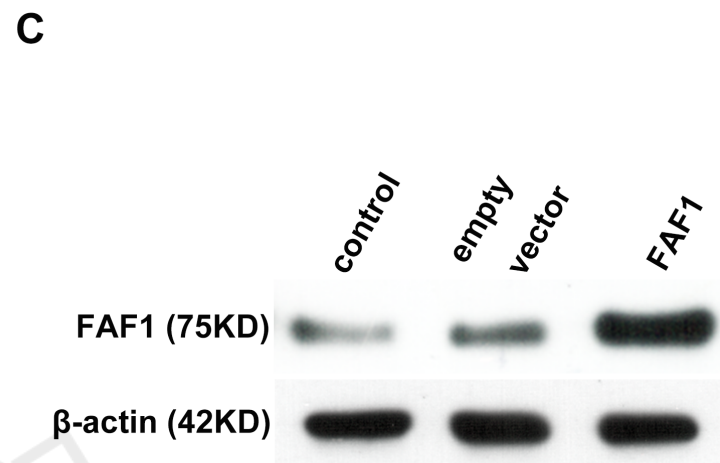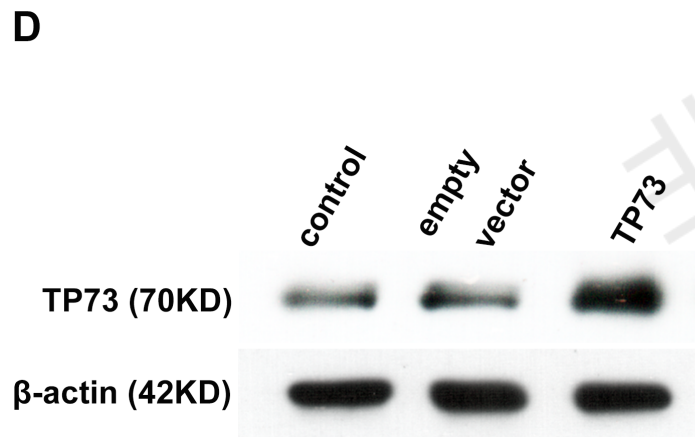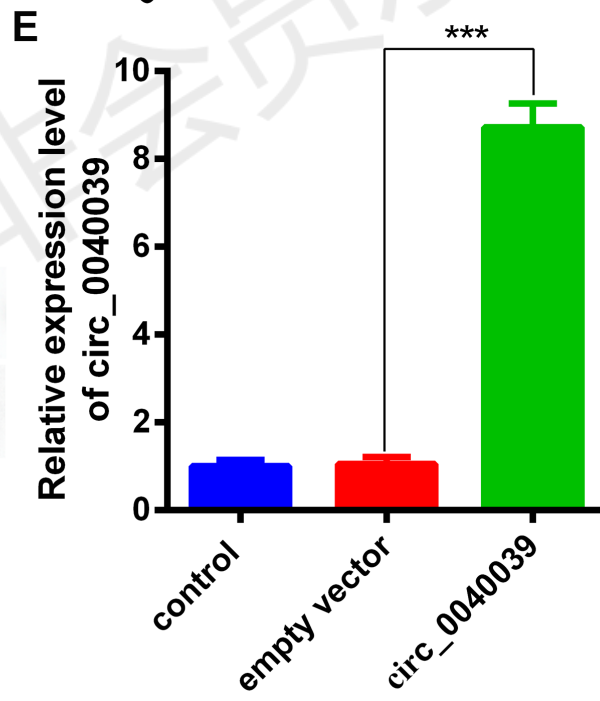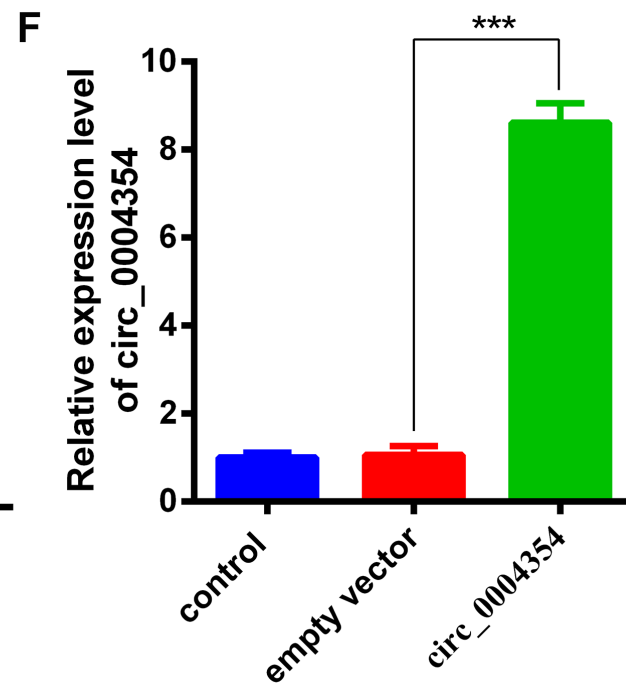

Supplement: Supplementary 6 — Supplementary Figure 3 Validation of the overexpression effect of circ_0040039, circ_0004354, FAF1, and TP73 in NPCs. The expression levels of FAF1 (A, C), TP73 (B, D), circ_0040039 (E), and circ_0004354 (F) were significantly increased in NPCs after transfected with the above overexpression vectors, respectively. ∗∗∗P <0.001. [file 2776440.f6.pdf]
